# From cheap entertainment to expensive pleasure: Cinema-going habits and motivations of young people in Türkiye

**DOI:** 10.1371/journal.pone.0350548

**Published:** 2026-06-05

**Authors:** Nermin Küçüksönmez, Abdullah Koçak, Nuh Akçakaya

**Affiliations:** 1 Faculty of Communication, Selçuk University, Konya, Turkey; 2 Department of Sociology, Selçuk University, Konya, Turkey; Universitas Mercatorum, ITALY

## Abstract

This study examines the cinema-going habits and motivations of university students in Türkiye from a class-based perspective. This study addresses a gap in the literature by examining cinema-going motivations in Türkiye through a class-based perspective. Participants (N = 1183) were recruited using a convenience sampling method from 12 universities across the NUTS 1 regions of Türkiye. This quantitative study employed three measurement tools to assess participants’ sociodemographic and socioeconomic characteristics, cinema-going habits, and motivations. The findings suggest that socialization was the most prominent factor associated with cinema-going motivation among participants. Cinema was not widely perceived by participants as a means of coping with loneliness, but rather as an activity associated with social interaction. The technical capabilities of cinema also emerged as an important factor associated with cinema attendance. While individuals reported different motivations, class-related differences were associated with variations in cinema-going behaviors within this sample. Participants with higher socioeconomic indicators tended to report higher levels of cinema attendance, whereas those with lower socioeconomic indicators reported lower levels of attendance. Given the convenience sampling design and the student-only composition of the sample, the findings should be interpreted with caution and should not be generalized beyond the study population.

## Introduction

The first film screening took place in 1895 at the Grand Café in Paris with the Lumière Brothers’ *The Arrival of a Train*. Although the small group of spectators initially felt uneasy—believing the train on the screen was coming toward them—they quickly formed a strong connection with cinema. Over time, cinema has acquired dedicated venues and established itself as an integral part of urban social life. In this process, the act of film viewing has come to involve not only the screen itself but also the spaces in which films are shown, leaving a lasting imprint on the audience’s memory [1]. This is because watching a film in a cinema involves its own unique set of conditions. Although it is a solitary act in some respects, the shared experience of watching the same content creates an invisible social group within the theater [2]. As such, cinema halls are not merely places for watching films; they are also social spaces that bring people together and foster interaction through collective cinematic experiences. In this sense, cinemas function as key cultural venues that play a formative and influential role in the process of socialization [3]. Viewed as an alternative public space, cinema extends the notion of spectatorship beyond conventional social and textual boundaries [4]. From this perspective, going to the cinema should be seen as a dynamic act—one shaped by specific rituals, driven by particular motivations, and influenced by broader social and economic conditions.

One of the main findings in research on the cinema-going experience is that it often revolves less around specific films and more around the broader experience surrounding the act of attending the cinema [5]. Cinema-going is motivated by a variety of desires, including curiosity about specific films and film stars, the pursuit of a cinematic experience, the need to balance the ordinary and extraordinary aspects of everyday life, social interaction, and the use of leisure time [5,6]. As in many parts of the world, over the years, the relationship between cinema and audiences and the experience of going to the cinema in Türkiye have evolved in line with changing social and economic conditions. The 1960s in Türkiye are often remembered as a time when cinema-going was seen as a “ceremonial” event, bringing together people from all walks of life and functioning as a place of social interaction [7]. In the post-2000 period, however, the cinema audience has shifted toward a younger, more educated, and socioeconomically privileged demographic [8,9].

In recent years, the field of cinema in Turkey has undergone significant change, driven by structural transformations in the broader media ecosystem. The rapid expansion of digital streaming platforms, particularly Netflix and Disney + , has increasingly pulled young audiences away from traditional cinemas. BluTV, once Turkey’s leading domestic platform, was acquired by Warner Bros. Discovery at the end of 2024 and, as of 2025, was integrated into the HBO Max/Max brand, marking a new phase of market consolidation. Industry reports indicate that as of 2023, Netflix had approximately 3.5 million active subscribers in Turkey, while BluTV maintained a strong local presence prior to its transition to Max. Meanwhile, according to official data from Turkish Statistical Institute [10], total cinema admissions reached 32.5 million in 2024—a figure that signals a partial yet insufficient recovery compared to the pre-pandemic period. Rising ticket prices, the increasing affordability and accessibility of digital platforms, and the difficulty cinemas face in regaining their pre-2019 momentum are among the key factors reshaping cinema-going habits. These developments not only transform established viewing practices but also redefine the cultural meanings and social functions attributed to the cinema experience.

Despite these transformations, existing academic work in Türkiye has not sufficiently examined how changing cinema practices intersect with class-based inequalities. Previous studies [8,9] have mostly focused on general audience behavior or technological change; however, research that systematically addresses the cinema-going experience within a theoretical and empirical framework grounded in class remains quite limited. Moreover, there are very few studies that analyze young people’s cinema habits using large-scale quantitative data. In particular, there is a notable lack of research that explicitly incorporates cultural capital and socioeconomic status into its analytical frameworks. These limitations point to a significant gap in the literature and underscore the need for research that critically interrogates the social stratification of cultural participation in today’s media environment.

Bourdieu’s theoretical approach [11] offers a productive foundation for addressing this gap, as it conceptualizes cultural consumption—including going to the cinema—not merely as a set of individual preferences but as a socially structured practice shaped by cultural capital, class position, and internalized habitus. Cultural capital has been widely discussed as a key factor shaping the value individuals place on cinema experiences, the meanings they attribute to cultural activities, and the frequency and form of their participation. Bourdieu’s concepts of distinction and the reproduction of inequalities provide a powerful framework for explaining how access to cultural activities such as cinema is unevenly distributed and how these inequalities reinforce existing social differences.

By integrating Bourdieu’s approach with contemporary audience dynamics, this study contributes to the literature by demonstrating that, cinema-going is shaped by the unequal distribution of cultural capital, yet film audiences are not homogeneous; the cinema experience must be situated within broader processes of social stratification. The study challenges common assumptions that film audiences are homogeneous and situates the cinema experience within broader processes of social stratification.

In this context, the present study examines the cinema-going habits and motivations of young people through the lens of class and cultural capital. It investigates the extent to which socioeconomic background influences attendance to cinema, the motivations behind choosing to attend cinemas, and the cultural structures shaping these practices. Understanding these dynamics is important not only for researchers studying cultural consumption and media audience behavior, but also for cinema exhibitors, digital platforms, and cultural policymakers seeking to adapt to a rapidly evolving media landscape. By offering empirical evidence on contemporary audience behavior, this study contributes to ongoing discussions about the future of cinema in Türkiye, the sustainability of cinemas, and the role of cultural policies in promoting equitable access to cultural spaces.

## Literature review

### From ceremony to ritual: Cinema-going habits

In the first half of the 20th century, cinema, along with radio, played a pivotal role in shaping media culture and became a key tool for producing mass popular culture. Ritualized practices emerged around cinema, and in this context, it contributed to shaping people’s daily routines and organizing their leisure time [12,13]. Empirical studies emphasizing that cinema-going became a habit and a routine [6,12,14–17] have largely focused on leisure time, interest, and preferences. These studies, which have examined the relationship between cinema and its audiences and sought to uncover what cinema has meant to people since the 1930s, have focused on different countries and national audiences. For instance, Kuhn [16], in her research on cinema culture in 1930s Britain, found that ordinary cinemagoers typically went to the movies two or three times a week with their family or friends and that their primary aim was to follow their favorite stars. For these cinemagoers, going to the cinema was simply part of everyday life, and it was regarded as an easy, social, enjoyable, and fondly remembered activity. Similarly, in her study on cinema culture, audience rituals, and habits in Slovenia, Pušnik [12] showed that up until the 1970s, cinematic practices were viewed as an inseparable part of social life. This view seems to persist today, with cinema continuing to serve as a means of relaxation and a reason for socializing or going on dates, thanks to the emotional experiences it offers such as fear, surprise, joy, and sorrow. Manning’s [18] findings further support this view, suggesting that cinema—through a long process of developing its own rituals, traditions, and customs—has come to be defined as a social habit.

In Türkiye, research on cinema-going habits and viewing experiences has accelerated especially since the 2010s [8,9,19,20]. Among these, Erkılıç’s [8] work stands out. Focusing on changes in cinema spaces and audience profiles, Erkılıç notes that in the 2000s, the audience profile of Turkish cinema has evolved into a “young and university-educated” demographic and that women have started to go to cinemas once again. A key reason for this is the perceived safety and convenience of shopping malls, which offered a variety of film options as well as consumption and leisure opportunities, making them appealing especially for families. Erkılıç also notes the growing popularity of at-home film viewing due to advancing technology. In a similar vein, Tanrıöver [9] characterizes cinema-going as a cultural practice of young, educated, urban individuals with higher socioeconomic status. Yüksel and Demir [21] argue that despite being seen as part of the digital generation, cinema continues to appeal to the 2000s cohort because of its functions as a space to socialize, to meet other young men and women, to see and be seen, and to temporarily escape real-world conditions—sometimes through behaviors like whistling, booing, or laughing during screenings.

Akbulut [22] approaches cinema-going experience in Türkiye from the 1960s to the 1980s through oral history. According to Akbulut, going to the cinema is not merely about watching films; it is a way of experiencing modern, urban citizenship. Cinema serves as a space for affiliating with a place, a time, an ideology, a class, or even with a mass beyond class and privilege—constituting a sociopolitical and cultural experience. Şanlıer and colleagues [23], on the other hand, have found that early on, cinemas were perceived as male-dominated spaces. However, with the rise of the nation-state and Turkish modernization, cinema was instrumentalized and became a space that also included women. According to Şanlıer and colleagues, between 1960 and 1980 in particular, cinema provided women with opportunities to leave home, socialize with other women, and experience the city in new ways. In this process, cinema helped establish a new public space and served as a site of freedom. Similarly, Gökmen and Gür [24] have found that open-air cinemas functioned not merely as film-viewing venues, but as spaces of social interaction. As emphasized directly or indirectly in many of these studies, cinema-going habits are shaped in parallel with broader processes of social change and transformation. Moreover, this pattern unfolds in similar ways both in Türkiye and globally. One of the most significant global indicators of this transformation today is the emergence of new viewing technologies. The rise of streaming services such as Netflix, Disney + , and Amazon Prime has introduced a widely accessible alternative to the traditional cinema experience. When rising ticket prices are added to this equation, the cinema-going experience has faced the risk of devaluation [25].

In recent years, structural changes within cinema environments have directly reshaped the motivations for going to the cinema. The emergence of shopping malls as dominant cinema venues—offering security, comfort and social visibility—has strengthened socially-oriented and identity-driven motivations, particularly among young and urban audiences [26]. Conversely, the widespread use of digital platforms and the convenience of home viewing have weakened ritualistic and habit-based motivations, while increasing motivations that transform cinema-going from a routine practice into a selective, occasional, event-focused activity grounded in the pursuit of novelty. This shift aligns with studies showing that attitudes toward digital technology are closely linked to motivations for going to the cinema [27]. Rising ticket prices further reinforce this trend by reducing the frequency of pleasure-driven viewing, increasing selectivity, and deepening class-based inequalities in cultural participation. Finally, the growing use of social media and the heightened influence of fear of missing out (FoMO) significantly amplify motivations centered on social capital, turning cinema-going into an activity intertwined with digital interaction and marked by high social visibility [28]. Taken together, these transformations demonstrate that changes in the broader media and economic context now actively shape the motivations for attending cinemas.

### Desire: Why do we want to go to the cinema?

Drawing on contemporary habit research, this study conceptualizes habit as a context-dependent and automatic behavioral pattern that emerges through repeated performances in stable environments. Motivation, by contrast, is treated as a goal-oriented and deliberate process that reflects individuals’ desires and intentions. Habit-based responses are triggered by situational cues and require relatively little conscious effort [29,30], whereas motivation involves reflective judgements about why an action—such as going to the cinema—should be carried out at a given moment [31]. In this sense, habit is a cue-driven, action-based process, while motivation corresponds to an internal desire shaped by goals and values. Accordingly, in this study, cinema-going habits and cinema-going motivations are treated as analytically distinct concepts that are related yet shaped by different psychological mechanisms.

One of the pioneering studies in the field is Stacey’s [32] research on Hollywood cinema and female audiences. The study categorizes the primary reasons women attended the cinema in the 1940s and 1950s under escape, identification, and consumerism. According to Stacey, the glamour and spectacle of Hollywood provided women with an avenue of escape. Through Hollywood films, female viewers constructed an alternative space that allowed them to escape both the hardships of wartime Britain and the traditional roles associated with British womanhood. Stacey redefines escapism in a positive light, framing it as a pleasurable aspect of cinema. This study is foundational in that it emphasizes how cinema-going goes beyond the mere enjoyment of ritualized events.

Moreover, the Uses and Gratifications Approach has played a significant role in explaining cinema-going motivations globally, and numerous studies have been conducted using this approach as their central theoretical basis (see: [33–39]). Research grounded in this approach highlights that cinema-going is partly driven by the pursuit of social gratification. Findings suggest that going to the cinema directly fosters social interaction and conversation [33,39]. According to Jarvie [2], although the cinema experience may involve minimal awareness of those seated nearby, a lack of direct social exchange during the film, and the possibility of participating without interacting with others, it still retains a social dimension. As such, it cannot be fully characterized as a solitary activity. On the contrary, cinema constitutes a social activity that may be experienced with family, school groups, friends, or romantic partners, and frequently serves as a source of conversation centered around films, stars, and related experiences. Recent studies examining the satisfactions derived from cinema focus on its emotional outcomes—namely hedonic pleasure and emotional gratification [40–42]. While reinforcing earlier findings on escapism and mood regulation, these studies also identify social benefits and relief from loneliness as key social gratifications and motivations for cinema-going [33,39]. A study by Tefertiller, Maxwell, and Morris [27] examines how social motivations—particularly social media factors—influence cinema-going behaviors. The study has explored how interactions on social media affect cinema-going frequency and how they intersect with other social motivations. In particular, the role of social capital and FoMO has been emphasized as significant in shaping individuals’ decisions to watch films in cinemas. In the context of Türkiye, while several studies have focused on measuring cinema-going habits, there has been no research to date specifically addressing the motivations behind cinema-going.

Building on these different motivational traditions, there is a need for a more integrative theoretical explanation to understand how individual desires interact with the socially structured nature of cinema-going. Bringing these perspectives together, this study draws on Bourdieu’s theory of cultural capital and the Uses and Gratifications (U&G) approach to explain how cinema-going practices are transformed within changing social and technological environments. Whereas Bourdieu emphasizes how class position and cultural capital shape access to cultural spaces, the value attributed to cinema, and the frequency of attendance, the U&G approach clarifies why individuals seek particular gratifications such as pleasure, social interaction, escapism, identity construction, or FoMO-driven social capital through cinema. Taken together, these two frameworks reveal that cinema-going motivations are not merely psychological preferences but also socially structured dispositions. Individuals’ capacity to obtain the gratifications they desire is determined by their cultural capital, economic resources, and the accessibility of alternative media environments.

This theoretical synthesis provides a useful framework for explaining how cinema-going motivations are reshaped by social and technological change. The rise of shopping malls as safe and controlled public spaces has turned the cinema into a site of social visibility and identity performance, strengthening social and identity-based motivations, particularly among young audiences [25]. The easy accessibility offered by digital platforms weakens ritualized and habitual cinema practices while encouraging more selective and event-oriented forms of participation [26]. In addition, social media dynamics and FoMO support young people’s tendency to view cinema-going as a means of producing social capital and maintaining cultural connections [27]. These contextual shifts demonstrate that motivations are not merely individual psychological preferences but social tendencies shaped by cultural capital, economic resources, and the opportunities afforded by media environments.

### From dream palace to consumer commodity

Cinema, a product of industrialization and urbanization that began in the Western world at the end of the 19th century, quickly became the most popular form of commercial leisure activity [12,18]. Providing both a social space and an experiential horizon for viewers, cinema served as an accessible form of entertainment for those unable to afford other leisure activities. For the poor, the uneducated, single women, and other members of the lower classes, cinema became the most affordable form of amusement. While theater and opera tickets were sold for one or two dollars, cinema tickets dropped to as little as 10–15 cents [4,43]. By the 1930s, these opulently decorated dream palaces that offered the cheapest and most accessible form of escape had become the primary mode of entertainment for many societies [44]. Over time, the changes and transformations in cinema have not been limited to technological advancements but have also encompassed broader socio-cultural shifts. Today, cinema is no longer perceived as an inexpensive form of entertainment; in fact, ticket prices now emerge as a major factor influencing audience decisions to go to the cinema [8,9,20,45].

Recent quantitative data from Turkey reveal the scale of this transformation more clearly. The national number of cinema-goers, which was around 59 million in 2019, fell by roughly 70% in 2020 due to COVID-19–related closures and was as low as 8.8 million in 2021. Although attendance partially recovered to approximately 31 million in 2023 and 32.5 million in 2024, these figures remain well below pre-pandemic levels and point not to a temporary disruption but to a long-term behavioral shift [10]. During the same period, average ticket prices also rose sharply: prices that were around 17 Turkish Lira (TL) in 2020 increased to 85 TL in 2023 and reached 200.34 TL in the first half of 2025 [46]. It is important to note that cinema ticket prices vary significantly across cities, venue types, and theater locations; therefore, the figures provided represent nationwide averages. This rapid increase in ticket prices has deepened class-based inequalities in access to cinema, pushed audiences toward more selective and event-focused attendance, and reduced the frequency of habit-based visits. These trends show that today’s economic pressures are reshaping not only who is able to go to the cinema in Turkey but also when, how, and with what motivations they choose to go.

At this point, Bourdieu’s concept of cultural capital becomes particularly relevant. Bourdieu [47] outlines three fundamental forms of capital: economic capital, which can be easily converted into money and property; social capital, which consists of social obligations and connections; and cultural capital, which exists in embodied, objectified, and institutionalized forms. Cultural capital can be defined as a set of cultural knowledge, institutionalized dispositions, and competencies [48,49]. According to Bourdieu [47], these forms of capital interact and reinforce one another. For example, economic capital may provide the means to develop a child’s cultural capital, which in turn is linked to future educational and professional success. In turn, educational attainment and career achievement can contribute to the accumulation of economic capital.

Objectified cultural capital refers to tangible cultural goods that can be used to gain advantage within a society. These may include clothing, art, books, or other physical objects that carry symbolic meaning and status within a cultural context. Objectified cultural capital is typically associated with higher social status. Moreover, interpreting or making sense of objectified forms of cultural capital requires a certain level of cultural literacy [50]. Bourdieu argues that the ability to appreciate art, or to possess a refined taste for it, is closely tied to one’s level of education and class status [51]. In contrast, for members of the lower class, meeting basic needs and securing survival are often the primary concerns [52]. In this context, cinema today can be considered a form of objectified cultural capital. Having moved away from its mission as inexpensive popular entertainment and now requiring a certain level of economic capital, cinema—particularly in the case of art films—also demands a certain level of education and social status from its audience. This situation highlights the class-based dimension of the cinema-going experience. Although individuals may have a certain degree of motivation to attend the cinema, they may lack the economic, cultural, or social means to do so. As a result, it becomes difficult to establish a consistent connection between the desire to go to the cinema and the actual practice of doing so.

In line with these dynamics, the study’s theoretical distinctions provide a basic framework for how cinema-going motivations and cultural capital will be measured in the empirical analysis. For this reason, it is necessary to clearly define the indicators through which the two main concepts of the research, namely cinema-going motivations and cultural capital, are operationalized.

In this study, cinema-going motivations are operationalized on the basis of the core dimensions of the Uses and Gratifications approach. Motivational domains such as pleasure, social interaction, aesthetic–technical appreciation, cultural enrichment, and escapism align with empirical patterns observed in young audiences’ cinema experiences and encompass both the experiential aspects and the social functions of cinema.

Cultural capital is measured across three distinct dimensions:

- embodied forms (interest in cinema, participation in artistic activities, film knowledge),
- objectified resources (frequency of cinema attendance, access to cinema venues, digital platform subscriptions),
- and institutionalized cultural indicators (parents’ level of education, students’ educational environment).

This framework makes the distinction between motivation and behavior visible by acknowledging that individuals may possess high motivation yet still be unable to attend the cinema regularly due to limited economic or cultural resources. In doing so, the study establishes an explanatory analytical foundation for examining class-based inequalities in cinema-going practices by linking theoretical concepts to concrete indicators.

## Methodology

Considering the empirical findings and theoretical orientations discussed above, it becomes clear that studies conducted in Türkiye have not sufficiently explored the desires and drives that lead audiences to go to the cinema. In this sense, a study that addresses the motivations and habits related to cinema-going would offer an original contribution to the existing literature. Additionally, Bourdieu’s [11] association of artistic activities with the upper classes stands in contrast to much of the literature on cinema audiences, which often regards cinema as a form of entertainment for the general public [16,17,32]. In this regard, whether cinema is a preferred cultural activity for the general public or for the upper classes should be tested empirically. Building on this premise, the present study addresses cinema-going habits and motivations from a class-based perspective and aims to address and clarify these contrasting arguments within the context of Türkiye.

This study was prepared based on the data obtained from the research project titled *“Cinema Despite Digital: The Behavior of Going to the Cinema Within the Context of Changing Audience Practices”*, project number 24401123, supported by the Selçuk University Scientific Research Projects Coordination Office and conducted under the supervision of Assoc. Prof. Dr. Nermin Küçüksönmez. The study was carried out with the approval of the Ethics Committee of Selçuk University Faculty of Communication, granted on 24.04.2024 under decision number 522.

The study was conducted online between October 22, 2024, and February 22, 2025. The survey, administered via Google Forms, targeted university students aged 18 and above. At the beginning of the questionnaire, participants were presented with an informed consent form, and their consent was obtained in writing. After confirming their voluntary participation in writing, the remaining questions were presented.

The unique standpoint outlined above necessitates the formulation of several research questions, which this study seeks to answer through a quantitative approach. These questions are as follows:

(1) What are university students motivations for going to the cinema?
(2) What are young people’s cinema-going habits?
(3) Do cinema-going habits have a class-based dimension?
(4) What are the key predictors of cinema-going habits and motivations?

The study aims to provide satisfactory answers to these questions through descriptive and correlational statistics. Each research question is addressed under one of the four sections in the findings chapter.

### Measures

To address the research questions, three different instruments—all developed by the researchers—were utilized:

#### Socioeconomic and sociodemographic information form.

This form includes categorical questions designed to capture participants’ socioeconomic and sociodemographic characteristics such as age, gender, parental education, family income, monthly expenses, high school location, and current university.

#### Cinema habits form.

This form includes both continuous and categorical variables that assess participants’ interest in cinema, frequency of cinema attendance, reasons for not going to the cinema, preference for domestic vs. foreign films, emotions prompting cinema attendance, meanings attributed to cinema, factors influencing the decision to go to the cinema, and preferred companions for cinema-going. All questions are designed to measure cinema habits based on participants’ actual behaviors.

#### Cinema-going motivation scale (CGMS).

The CGMS was developed specifically for this study. An initial pool of 25 items was created for the scale, and five experts were consulted to assess content validity. Expert evaluations were analyzed using Lawshe’s (1975) [53] technique, and items that did not meet the required Content Validity Ratio were removed, reducing the item pool to 23. A preliminary test was then conducted for the 23-item questionnaire. The psychometric properties of the scale were examined across two studies, including the pre-test and the main test.

The pre-test was carried out face-to-face at Selçuk University using convenience sampling (n = 119). In the pre-test, the factor structure of the CGMS was explored using Principal Components Analysis (Σλ = 73.1%; KMO Measure of Sampling Adequacy = .869; Bartlett’s Test of Sphericity: χ² = 1791.939; p < .001). Cronbach’s alpha internal consistency coefficients were also examined and found to range between.830 and.923. Validity and reliability tests for the CGMS were repeated in the main study, where the primary data were collected, providing additional evidence. The same four-factor structure consisting of 17 items identified in the pre-test was replicated (Σλ = 74.9%).

In the main study, a Confirmatory Factor Analysis (CFA) was also conducted using the maximum likelihood method (p < .001). With the exception of the χ²/df ratio (6.741), all fit indices indicated excellent or near-excellent levels (RMSEA = .070; NFI = .951; AGFI = .933; TLI/NNFI = .945; CFI = .958; GFI = .933). The elevated χ²/df value is attributable to the large sample size (N = 1183), as commonly noted in the literature [54]. The CFA provided additional evidence supporting the construct validity of the scale.

To assess internal consistency further, item–total correlations were examined. All items showed statistically significant correlations (p < .001), ranging from.436 to.770, indicating satisfactory internal consistency. Across both pre-test and main-test, the reliability analyses demonstrated that the CGMS possesses strong psychometric qualities.

The final version of the scale consists of 17 items rated on a 5-point Likert scale, measuring both general cinema-going motivations (α = .922) and specific motivational dimensions, including Appeal of Cinema (α = .888), Desire for Cultural Enrichment (α = .940), Technical Features of Cinema (α = .893), and Escape from Problems (α = .758).

### Participants and procedure

The research data were collected online from students at 12 different universities located in 12 cities across Türkiye’s Level 1 regions, as defined by the Nomenclature of Territorial Units for Statistics (NUTS 1). With a 95% confidence level and a 3% margin of error, the required sample size was determined to be sufficient for statistical estimation within the study sample; however, the use of convenience sampling limits the generalizability of the findings. (N = 7,081,289) [55]. However, during the data collection process, the target number of 1,068 participants was exceeded and data were ultimately collected from 1,183 individuals. The universities, selected using a convenience sampling method, included student populations from upper, middle, and lower social classes. It is observed that universities which charge tuition fees or admit students with high academic standards (for example, Istanbul University, Medipol University) tend to have a student profile drawn predominantly from higher socio-economic backgrounds. In contrast, universities that provide free education and admit students with moderate levels of academic achievement (such as Selçuk University and Akdeniz University) generally cater to students from middle socio-economic backgrounds. Similarly, universities offering free education and admitting students with lower academic scores (for instance, Aksaray University and Kırklareli University) are observed to serve a student population largely from lower socio-economic backgrounds. These varying structural characteristics lead universities to host students from different social classes. Convenience sampling has limitations in terms of generalizability; however it is frequently employed in studies operating under financial constraints [56]. Although the selected universities may not reflect all characteristics of the population precisely, their diversity in terms of class composition and geographic distribution across 12 different regions provides a diverse empirical basis for identifying patterns within the study sample, although the findings cannot be generalized beyond it.

After the data were collected, normality tests were conducted and the assumptions of the statistical analyses were checked. Depending on the distributional characteristics of the data, Pearson correlation, Spearman correlation, one-way ANOVA, t-test, chi-square test, cluster analysis, multiple linear regression and ordinal regression analyses were performed using SPSS 27.

## Results

The participant profile shows considerable variation in terms of gender, geographic distribution, and socioeconomic background, reflecting a diverse student sample drawn from different regions of Türkiye. A total of 1,183 students from 12 different universities across the country participated in this study. Of these participants, 52.1% were female (*n* = 616) and 47.9% were male (*n* = 567). The average age was 21.21 years (*SD* = 2.51). Participants were recruited from the following universities, all located in NUTS 1 regions: Selçuk (12.9%), Akdeniz (12%), İstanbul Medipol (11.8%), Atatürk (10.1%), Anadolu (9.7%), Kırklareli (8.2%), İnönü (7.6%), Gaziantep (7.4%), Afyon Kocatepe (7.3%), Trabzon (4.7%), Samsun (4.3%), and Aksaray (4%). Regarding monthly personal spending, 30.1% of participants reported spending 5,000 TL or less (lowest category), while 11.1% reported spending 20,000 TL or more (highest category). In terms of family income, 10.3% of participants fell into the lowest income bracket (15,000 TL or less), and 9.6% were in the highest bracket (above 105,000 TL). For paternal education levels, 1.9% of fathers were literate only, 17.5% had completed elementary school, 14.8% had completed middle school, 46.5% had completed high school, 16.6% held a university degree, and 2.8% had a postgraduate degree. For maternal education levels, 6.8% of mothers were literate only, 33.1% had completed elementary school, 19.3% had completed middle school, 23.7% had completed high school, 15% held a university degree, and 2.2% had a postgraduate degree. In terms of the location where participants completed high school, 31% studied in a district town, 22.2% in a provincial city, 26.4% in a metropolitan area, and 20.4% in a major metropolis (Istanbul, Ankara, or Izmir).

The participants display a balanced profile in terms of gender distribution; however, it is difficult to argue that this balance is reflected similarly across the universities. When the regions and cities in which the universities are located are taken into account, clear variations within the sample become apparent. More detailed information on the sociodemographic characteristics of the participants is presented in Table 1.

**Table 1:** Sociodemographic information of the participants.

Gender	F	%	University – Region – City	F	%
Female	616	52,1	Selçuk U. - Western Anatolia – Konya	153	12,9
Male	567	47,9	Akdeniz U. - Mediterranean – Antalya	142	12,0
Total	1183	100,0	İstanbul Medipol U. - Marmara – İstanbul	140	11,8
**Monthly Average Personal Expenditure**	F	%	Atatürk U. - Northeastern Anatolia – Erzurum	119	10,1
5.000 TL and below	356	30,1	Anadolu U. - Eastern Marmara – Eskişehir	115	9,7
5.000-10.000 TL	451	38,1	Kırklareli U.- Western Marmara – Kırklareli	97	8,2
10.000- 15.000 TL	168	14,2	İnönü U. - Central Anatolia – Malatya	90	7,6
15.000-20.000 TL	77	6,5	Gaziantep U. - Southeastern Anatolia – Gaziantep	87	7,4
20.000-25.000 TL	38	3,2	Afyon Kocatepe U. - Aegean – Afyon	86	7,3
25.000 TL and above	93	7,9	Trabzon U. - Eastern Black Sea – Trabzon	56	4,7
Total	1183	100,0	Samsun U. - Western Black Sea – Samsun	51	4,3
**Approximate Family Income**	**F**	**%**	Aksaray U. - Central-East Anatolia – Aksaray	47	4,0
15.000 TL and below	122	10,3	Total	1183	100,0
15.000-30.000 TL	287	24,3	**Father’s Education Level**	**F**	**%**
30.000-45.000 TL	223	18,9	Literate (no formal schooling)	22	1,9
45.000- 60.000 TL	164	13,9	Elementary school	207	17,5
60.000- 75.000 TL	121	10,2	Middle school	175	14,8
75.000-90.000 TL	84	7,1	High school	550	46,5
90.000- 105.000 TL	68	5,7	University degree	196	16,6
105.000- 120.000 TL	24	2,0	Postgraduate degree	33	2,8
120.000- 135.000 TL	24	2,0	Total	1183	100,0
135.000 TL and above	66	5,6	**Mother’s Education Level**	**F**	**%**
Total	1183	100,0	Literate (no formal schooling)	81	6,8
**Place where high school was completed**	**F**	**%**	Elementary school	391	33,1
District town	367	31,0	Middle school	228	19,3
Provincial city	263	22,2	High school	280	23,7
Metropolitan area	312	26,4	University degree	177	15,0
Major metropolis (İstanbul, Ankara, İzmir)	241	20,4	Postgraduate degree	26	2,2
Total	1183	100,0	Total	1183	100,0

### Cinema-going habits

97% of the participants reported having gone to the cinema at least once in their lives, while only 3% (*n* = 36) stated that they had never been to the cinema before. This indicates that cinema remains a common form of entertainment and socialization for the young participants in this sample. When asked about the frequency of cinema attendance over the past 2–3 years, 46.4% said they had gone 1–3 times, 21.6% had gone 4–6 times, 10.7% had gone 7–10 times, and 6.5% had gone more than 11 times. Meanwhile, 14.9% reported not attending the cinema at all during this period. Participants were also asked to rate their level of interest in cinema on a 10-point scale. The mean score was 5.63, with a standard deviation of 2.16. Responses to the question of who they go to the cinema with suggest that one’s social environment plays a key role in this activity: 50.4% reported going with friends, 22.5% with a boyfriend or girlfriend, 15% with family, and 12.1% said they go alone. Participants also reported a variety of emotional states as reasons for attending the cinema: 50.9% said they went when feeling happy, 31.5% when bored, 7.6% when feeling depressed, 6.4% when sad, and 3.6% when tired. Responses to what cinema-going means to participants also revealed a broad range of responses. The most frequently reported meaning was a social activity (25.7%), followed by entertainment (20.4%), a passing time activity (19.3%), a hobby (11.1%), an escape (9.8%), and a way of life (4.5%). This diversity suggests that cinema holds different meanings for individuals but is generally associated with positive emotional states and social engagement.

When participants’ film-viewing habits were examined, a strong preference for digital platforms emerged. 38.1% reported watching films both in cinemas and on other platforms, 26.2% primarily used the internet, and 20.5% preferred digital streaming platforms. Only 8.6% (*n* = 102) stated that they usually watched films in cinemas. This suggests that, among the participants, while cinema is still valued, digitalization appears to have taken the lead. Reasons for not attending the cinema included the high cost of tickets (35.1%), the growing popularity of digital platforms (32.5%), and a general loss of the habit of cinema-going (9.9%). These findings show that economic and technological factors associated with interest in cinema. On a 5-point Likert scale, participants reported that the factors most associated with their decision to go to the cinema were film trailers ($\bar{x}$ = 3.46), friends ($\bar{x}$ = 3.37), social media ($\bar{x}$ = 3.37), the internet ($\bar{x}$ = 3.35), IMDb scores ($\bar{x}$ = 3.07), posters ($\bar{x}$ = 2.71), and cinema program listings ($\bar{x}$ = 2.60).

### Cinema-going motivations

While the data above offer some insights into participants’ cinema-going habits, motivations for going to the cinema require a broader and more in-depth assessment. In this context, some descriptive statistics related to the Cinema-Going Motivation Scale (CGMS) help clarify participants’ intrinsic motivations. As shown in Table 2, the overall score on the CGMS is at a moderate level ($\bar{x}$ = 3.31, SD = 0.840). Similarly, in a separate question that asked participants to rate their general interest in cinema on a scale from 0 to 10, the average score was slightly above moderate ($\bar{x}$ = 6.06, SD = 2.37). This suggests that motivation and interest in cinema remain present among the participants, albeit at moderate to low levels.

**Table 2:** Correlations between the CGMS and factors influencing the decision to go to the cinema.

		1	2	3	4	5	6	7	8
1	CGMS	1.000							
2	Friends’ influence	.175^*^	1.000						
3	Internet	.163^**^	.229^**^	1.000					
4	Social media	.183^**^	.261^**^	.748^**^	1.000				
5	IMDb scores	.154^**^	.185^**^	.357^**^	.396^**^	1.000			
6	Posters	.222^**^	.100^**^	.239^**^	.302^**^	.421^**^	1.000		
7	Cinema programs	.229^**^	.108^**^	.220^**^	.211^**^	.314^**^	.517^**^	1.000	
8	Trailers	.199^**^	.199^**^	.248^**^	.283^**^	.332^**^	.438^**^	.399^**^	1.000

Looking at the subdimensions of the CGMS in Table 2, the specific motivations that constitute general motivation are ranked as follows: technical features of cinema, appeal of cinema, desire for cultural development, and escape from problems. This indicates that both technical features and related aesthetic appeal are the most significant factors associated with cinema-going motivations among the participants. When individual items in Table 3 are examined, the most prominent motivation appears to be spending time with friends. Interestingly, the least influential factor is loneliness. This suggests that cinema is perceived primarily as a social activity, rather than as a way to cope with solitude. Attending the cinema is therefore seen not as an escape from loneliness, but as an opportunity to enjoy companionship.

**Table 3:** Descriptive Statistics of the Cinema-Going Motivation Scale (CGMS).

Cinema-Going Motivation Scale (CGMS)	N	$\bar{x}$	ss
I enjoy spending time at the cinema with people I love.	1116	3.95	1.053
I enjoy talking about movies I watch at the cinema with my friends.	1117	3.83	1.101
I enjoy watching movies in the ambiance of the cinema hall.	1101	3.83	1.142
I enjoy watching movies on the big screen with high-quality image and sound technology.	1100	3.76	1.144
I think it is easier to focus on the movie in the dark environment of the cinema hall.	1095	3.75	1.202
Watching movies in the cinema entertains me a lot.	1112	3.68	1.073
I feel good while watching movies in the cinema.	1130	3.66	1.081
I think 3D movies are more effective and satisfying in the cinema.	1092	3.51	1.275
Watching movies in the cinema has always relaxed me.	1104	3.45	1.121
I feel rested while watching movies in the cinema.	1103	3.35	1.156
I go to the cinema because I think it is an important cultural-artistic activity.	1099	3.15	1.249
I think going to the cinema increases my general cultural knowledge.	1087	3.13	1.234
I think going to the cinema improves my aesthetic understanding.	1088	3.01	1.263
Going to the cinema helps me forget my problems.	1101	2.98	1.270
I think going to the cinema increases my taste in art.	1092	2.95	1.271
I have not adopted other ways of watching films.	1081	2.22	1.184
I go to the cinema because I feel lonely.	1089	2.21	1.228
Technical Features of Cinema	1119	3.69	1.055
Appeal of Cinema	1157	3.63	0.912
Desire for Cultural Development	1107	3.06	1.155
Escape from Problems	1107	2.48	1.016
Overall Cinema-Going Motivation Scale (CGMS)	1166	3.31	0.840

Participants who typically go to the cinema with a romantic partner ($\bar{x}$ = 3.42) scored higher on the CGMS than those who go alone ($\bar{x}$ = 3.30). Similarly, those who view cinema as a way of life ($\bar{x}$ = 3.73) had higher CGMS scores than those who see it merely as a means of passing time ($\bar{x}$ = 3.31). Furthermore, participants who perceive cinema as a way of life reported going to the cinema in all emotional states, whereas those who assign other meanings to cinema were more likely to go only when feeling happy or bored (*χ²* = 372.237, *p* < .05). Likewise, those who consider cinema a way of life reported going with various types of companions, while those with other interpretations of cinema were more likely to go only with friends (excluding romantic partners) (*χ²* = 190.330, *p* < .05). Similarly, individuals who go to the cinema when happy or bored were more likely to attend with friends (*χ²* = 148.874, *p* < .05).

As expected, participants who watch films primarily on platforms other than the cinema (*F* = 1.417, *p* = .002), those who had never attended the cinema (*t* = 5.168, *p* < .001), individuals whose fathers (*F* = 1.294, *p* < .05) or mothers (*F* = 1.262, *p* < .05) have lower education levels, and male participants (*t* = 3.306, *p* < .001) had lower CGMS scores.

While all decision-making factors regarding cinema-going were found to be related to CGMS scores, the weakest correlation was observed with IMDb ratings (*r* = .154, *p* < .001), whereas the strongest correlation was cinema program listings (*r* = .229, *p* < .001). This indicates that participants with a more cultural orientation tend to have higher motivation scores. Indeed, participants who reported artistic engagement as their primary reason for watching films (*r* = .243, *p* < .001) or who prefer to watch films in cinemas rather than through other mediums (*r* = .321, *p* < .001) also had significantly higher CGMS scores

### The class dimension of cinema-going

While the CGMS provides insights into motivations for cinema-going, further analyses were conducted to explore which groups show a greater inclination toward cinema attendance, independent of motivation. This is because it is possible to encounter individuals who have the motivation but lack the means to attend. To categorize cinemagoers into distinct groups, a cluster analysis was conducted based on responses to four questions related to cinema-going tendencies. These questions were: (1) Have you ever been to the cinema in your life?, (2) How many times have you been to the cinema in the last 2–3 years?, (3) What are your cinema-going and film-viewing habits?, and (4) What are your reasons for not going to the cinema?

Cluster analysis was conducted using the TwoStep algorithm, which is capable of handling both categorical and continuous variables simultaneously. Four variables were included in the analysis, and a three-cluster structure was obtained. To assess the quality of cluster separation, the average silhouette coefficient was examined. This value was calculated as 0.20, which is classified as “fair” by SPSS. This finding indicates that the clusters are not highly distinct, but they possess an analytically acceptable level of separation. The number of clusters was determined using the automatic model selection procedure of the TwoStep algorithm, which calculates the optimal number of clusters based on the Bayesian Information Criterion (BIC). Based on comparisons of BIC values, the three-cluster solution was identified as the most appropriate model.

The cluster analysis, which yielded a silhouette coefficient of 0.20, indicating an analytically acceptable level of cluster separation, identified three main groups encompassing 78% of participants, while 22% did not fit into any cluster. Among the variables used, the number of cinema visits in the last 2–3 years was the most important factor distinguishing these groups. The primary reason why not all participants could be included in the analysis is missing data in the variables used. Specifically, the question “What are your reasons for not going to the cinema?” was answered only by those who do not go to the cinema or whose attendance frequency has decreased, while participants who go to the cinema regularly left this question blank. Since the TwoStep algorithm automatically excludes observations with missing data on any variable, 260 participants who did not answer this question could not be included in the cluster analysis.

**Fig 1:**
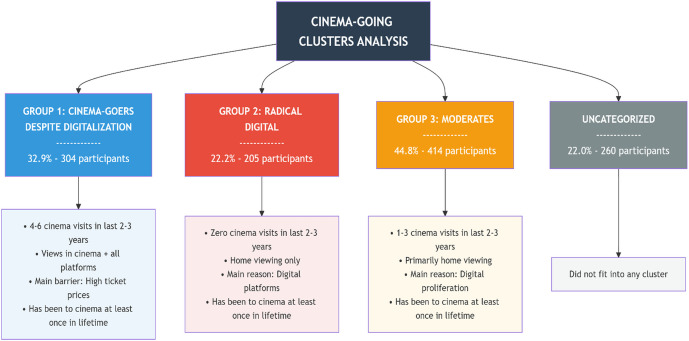
Cinema-going tendencies by cluster.

When Fig 1 is examined, it can be seen that within the participant population there are those who fall into a cluster and those who do not. In this sense, Fig 1 reflects the relatively dominant characteristics of the three emerging groups and provides important insights into each group’s relationship with cinema. However, the labels assigned to the groups in the figure represent only the dominant tendencies, and group members may also exhibit different inclinations. The fact that these descriptions and labels reflect general tendencies, while also allowing for within-group diversity, can be seen more clearly in Table 4.

**Table 4:** Component distributions of cinema-going tendency clusters.

	Cinema-goers despite digitalization (n = 304)	Radical digitalizers (n = 205)	Moderates (n = 414)
**How many times have you attended the cinema in recent years (last 2–3 years)?**			
Never attended	3,4%	71,4%	0,0%
1–3 times	0,0%	26,0%	100,0%
4–6 times	62,2%	2,6%	0,0%
7–10 times	22,4%	0,0%	0,0%
11 or more	11,9%	0,0%	0,0%
Total	100,0%	100,0%	100,0%
**What are your cinema-going and film-viewing habits?**			
I do not attend cinema; I mostly watch films on television	0,0%	24,5%	0,0%
I do not attend cinema; I mostly watch films on the internet	16,4%	39,8%	36,2%
I do not attend cinema; I mostly watch films on DVD	0,7%	3,1%	0,0%
I do not attend cinema; I mostly watch films on digital platforms	15,7%	16,8%	31,4%
I attend cinema; I mostly watch films in cinema	11,6%	14,8%	0,00%
I attend cinema; I watch films both in cinema and on digital platforms, the internet, or DVD	55,8%	1,0%	32,4%
Total	100,0%	100,0%	100,0%
**What are your reasons for not attending the cinema? (If applicable)**			
I have lost the habit of going to the cinema	8,8%	18,4%	12,0%
The proliferation of digital platforms	37,4%	40,8%	44,4%
The high cost of cinema tickets	53,4%	35,7%	42,1%
Other	0,3%	5,1%	1,5%
Total	100,0%	100,0%	100,0%
**Have you ever attended the cinema in your life?**			
Never attended	0,0%	13,8%	0,0%
Yes, I have attended	100,0%	86,2%	100,0%
Total	100,0%	100,0%	100,0%

Among those included in the clusters, the first group, comprising 32.9% of participants (n = 304), tended to have attended the cinema 4–6 times in the past 2–3 years and tended to watch films both in cinemas and on other platforms. They most frequently attributed their recent reduction or cessation of cinema attendance to the high cost of tickets (53.4%), followed by the proliferation of digital platforms (37.4%). This group, which showed the highest cinema attendance frequency in the sample, can be described as “cinema-goers despite digitalization”.

The second group, representing 22.2% (n = 205), consisted of individuals the majority of whom (71.4%) had not attended the cinema at all in the last 2–3 years, while 26.0% had attended only 1–3 times. They tended to watch films primarily at home but had attended the cinema at least once in their lifetime. This group most frequently attributed their recent non-attendance to the rise of digital platforms (40.8%) and can be labeled as “radical digitalizers”. The third group, making up 44.8% (n = 414), tended to have attended the cinema 1–3 times in the last 2–3 years.

Similar to the second group, they most frequently cited the proliferation of digital platforms (44.4%) and the high cost of tickets (42.1%) as their reasons for reduced cinema attendance and tended to watch films primarily at home. This group can be described as “moderates”.

More detailed descriptive information regarding each group’s relationship with cinema is provided in Table 4. As such, Table 4 offers a detailed reflection of the key tendencies within the groups. Nevertheless, the table also reveals that these group categorizations lack rigid boundaries and that the groups are, to some degree, interpenetrable.

Although some participants maintain a distance from the cinema, there are individuals who have not completely abandoned cinema despite the rise of digital platforms. Indeed, the cluster analysis revealed that while one group exhibited a strong inclination toward cinema attendance, another group showed a weaker yet still observable tendency. The average CGMS scores were 3.41 for the “cinema-goers despite digitalization”, 3.26 for the “moderates”, and 3.03 for the “radical digitalizers” (*r*_spearman_ = .153, *p* < .05). Despite variations in cinema-going habits, motivation levels did not differ significantly. This suggests that individuals generally possess some degree of motivation toward cinema regardless of cinema attendance. However, not everyone attends the cinema with the same regularity or has the opportunity to do so, indicating that factors beyond motivation are associated with cinema attendance. To investigate whether cinema-going behaviors are linked to class realities, a second cluster analysis was conducted. In the second cluster analysis, in addition to cinema-going tendencies, five other variables were included: (1) family income, (2) monthly personal expenditure, (3) mother’s education, (4) father’s education, and (5) university attended. As in the first analysis, the TwoStep algorithm—capable of handling both categorical and continuous variables simultaneously—was used. The cluster membership obtained from the first analysis was also included as a new variable, resulting in a total of six variables. The analysis yielded a two-cluster structure. (The number of clusters was determined using the automatic model selection procedure of the TwoStep algorithm, which calculates the optimal number of clusters based on the Bayesian Information Criterion (BIC). Based on comparisons of BIC values, the two-cluster solution was identified as the most appropriate model.) Cluster separation quality was assessed using the average silhouette coefficient, which was calculated as 0.2. This value corresponds to a ‘fair’ level according to the SPSS classification, indicating that the separation between clusters is limited yet analytically acceptable. As in the first analysis, missing data prevented the inclusion of all participants; therefore, the cluster analysis was again conducted with only the 923 participants who responded to the relevant questions, while the remaining 260 participants were excluded. When examining the discriminatory power of the variables, family income, monthly personal expenditure, and tendency to go to the cinema were found to be the variables most strongly contributing to cluster separation, in that order, followed by mother’s education, father’s education, and the university attended. This finding suggests that the clustering was shaped primarily around economic and consumption-based variables.

**Fig 2:**
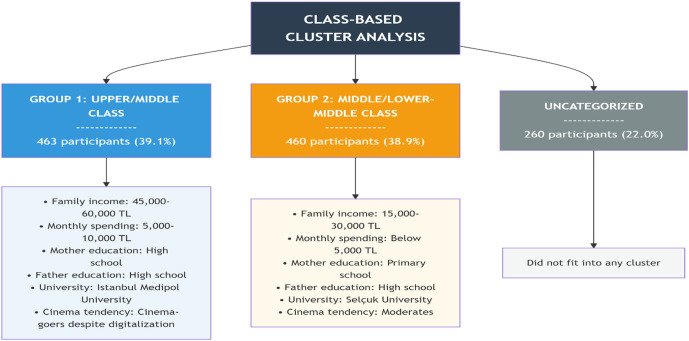
Class-based clusters.

The two clusters that emerged from the analysis were observed to reflect certain class-related characteristics. The first group displayed tendencies toward relatively higher income and expenditure levels. Participants in this group tended to have family incomes between 45,000 and 60,000 TL, monthly expenditures between 5,000 and 10,000 TL, parents who were mostly high school graduates, and were enrolled in a private foundation university (İstanbul Medipol). However, it should be noted that these characteristics are not definitive for all group members, and as Fig 2 shows, different profiles can also be found within the group. This group, exhibiting upper- or middle-class tendencies, largely corresponds to the “cinema-goers despite digitalization” cluster.

The second group, in contrast, displayed tendencies toward lower income and expenditure levels (Fig 2). Participants in this group tended to have family incomes between 15,000 and 30,000 TL, monthly expenditures below 5,000 TL, mothers with mostly primary education, fathers with mostly high school education, and were enrolled at Selçuk University. Nevertheless, these characteristics do not have sharp boundaries, and diversity within the group should be acknowledged. This group aligns with the ‘moderates’ and reflects middle or lower-middle class tendencies.

The clustering of these two groups in terms of class-related characteristics suggests that, within this sample, cinema attendance appears to be higher among individuals with upper- and middle-class socioeconomic indicators within this sample. However, it should be emphasized that these findings represent dominant patterns rather than a definitive class divide, and that within-group diversity exists. Given that class is measured in this study through indirect indicators such as income, parental education, and university type, interpretations should be made cautiously. As shown in Table 5, participants from various income, expenditure, and education levels are present in both groups, and the descriptive categories presented reflect only the dominant characteristics of each group. Therefore, the findings should be interpreted not as definitive class-related patterns, but as class-related tendencies or class-related patterns observed within this sample.

**Table 5:** Component distributions of class-based clusters.

	Upper- or Middle-Class Tendencies	Middle or Lower-Middle Class Tendencies
**Family income (approximate monthly income of household)**		
15.000 TL and below	1,1%	21,0%
15.000–30.000 TL	6,9%	45,7%
30.000–45.000 TL	17,4%	17,9%
45.000–60.000 TL	21,6%	6,8%
60.000–75.000 TL	18,3%	3,6%
75.000–90.000 TL	11,8%	2,7%
90.000–105.000 TL	9,6%	0,2%
105.000–120.000 TL	2,9%	0,7%
120.000–135.000 TL	2,0%	0,5%
135.000 TL and above	8,5%	0,9%
Total	100,0%	100,0%
**Monthly personal expenditure?**		
5.000 TL and below	8,7%	56,3%
5.000–10.000 TL	41,0%	35,7%
10.000–15.000 TL	22,9%	2,7%
15.000–20.000 TL	10,0%	1,6%
20.000–25.000 TL	4,7%	1,4%
25.000 TL and above	12,7%	2,3%
Total	100,0%	100,0%
**Mother’s education level**		
Literate	0,9%	14,0%
Primary school	17,8%	50,5%
Middle school	19,6%	17,9%
High school	32,5%	14,5%
University	24,7%	2,7%
Postgraduate	4,5%	0,5%
Total	100,0%	100,0%
**Father’s education level**		
Literate	0,0%	3,6%
Primary school	6,5%	30,5%
Middle school	16,7%	12,9%
High school	46,1%	49,8%
University	25,2%	2,9%
Postgraduate	5,6%	0,2%
Total	100,0%	100,0%
**University attended**		
İstanbul Medipol University	20,9%	2,5%
Kırklareli University	5,8%	9,7%
Afyon Kocatepe University	2,4%	11,3%
Anadolu University	15,4%	3,4%
Selçuk University	11,1%	17,2%
Akdeniz University	15,6%	9,5%
İnönü University	2,7%	10,9%
Samsun University	5,8%	3,4%
Trabzon University	2,4%	9,0%
Atatürk University	10,2%	7,7%
Aksaray University	2,0%	5,9%
Gaziantep University	5,6%	9,5%
Total	100,0%	100,0%
**Cinema-going tendency cluster**		
Radical Digitalizers	6,0%	38,2%
Moderates	44,3%	45,7%
Cinema-goers despite digitalization	49,7%	16,1%
Total	100,0%	100,0%

The CGMS scores also varied by class cluster, with the middle-class tendency group scoring lower ($\bar{x}$ = 3.21) than the higher socioeconomic status tendency group ($\bar{x}$ = 3.31). Furthermore, as indicated in Table 6, class-related variables—mother’s education (*r*_spearman_ = .256), father’s education (*r*_spearman_ = .142), family income (*r*_spearman_ = .201), and monthly expenditure (*r*_spearman_ = .165)—were significantly correlated with CGMS scores (*p* < .05).

**Table 6:** The relationship between the CGMS and class-related characteristics.

	CGMS	Mother’s education	Father’s education	Family income	Monthly expenditure
CGMS	1.000				
Mother’s education	.256^**^	1.000			
Father’s education	.142^**^	.542^**^	1.000		
Family income	.201^**^	.334^**^	.270^**^	1.000	
Monthly expenditure	.165^**^	.184^**^	.144^**^	.560^**^	1.000

### Predictors of cinema-going motivations and habits

Although many variables are correlated with the CGMS, some continuous variables show relatively stronger associations than others. Accordingly, cinema-going motivation is associated with the level of interest in cinema (X_1_) and by the extent to which social media (X_2_) and film posters (X_3_) are linked to the decision to go the cinema. Additionally, going to the cinema solely for artistic purposes (X_4_), considering film watching as a means of relaxation (X_5_), discussing films with one’s social circle (X_6_), and preferring the cinema as a film-watching venue despite other available platforms (X_7_) show statistically significant associations with cinema-going motivation. Based on all these variables, a multiple linear regression model was constructed. Rather than relying on strong, pre-established theoretical predictions, the model was developed to identify data-driven patterns that may assist in formulating hypotheses for future research. In this context, the analysis focused on the statistical significance of the relationships, with the findings presented as a foundation for subsequent theoretically guided confirmatory studies. The data-driven selection of independent variables is preferred in cases where no established theoretical model exists in the field, as it helps guide hypotheses for future investigations. To evaluate the validity of the regression model, assumption checks were conducted, and these checks indicated that the model met the required conditions. In the normality assessment, both the normal probability plot (P–P Plot) and the histogram of standardized residuals visually confirmed that the distribution closely approximated a normal curve. The assumptions of linearity and homoscedasticity were confirmed, as the scatterplot of standardized residuals against standardized predicted values showed points randomly dispersed around zero without any discernible pattern. This visual finding was also statistically supported by the Breusch–Pagan test (χ²(7) = 0.000, p > .05), which confirmed that the assumption of constant variance had not been violated. Additionally, the Durbin–Watson statistic of 1.968 indicated that no autocorrelation was present among the residuals. Regarding multicollinearity, tolerance values ranged between 0.748 and 0.887 (VIF: 1.127–1.369) for all independent variables, demonstrating that multicollinearity was not a concern. Taken together, these diagnostic analyses support the validity and reliability of the regression model.

Table 7 presents the results of a multiple linear regression model assessing the relationship between the CGMS as the dependent variable and several independent variables hypothesized to have relatively strong associations with it. The model summary shows an R (correlation coefficient) of 0.595, indicating a moderate relationship between the dependent and independent variables. The adjusted R² value of 0.350 demonstrates the model’s goodness of fit and suggests that the independent variables collectively explain 35% of the variance in CGMS scores. The constant term in the model is 1.14, representing the expected value of CGMS when all independent variables are zero. Examination of the regression coefficients (B) reveals that all identified independent variables have positive and statistically significant associations with CGMS, indicating that these variables are positively related to cinema-going motivation. The first predictor in the model is the level of interest in cinema (_X1_). According to the model, a one-unit increase in cinema interest corresponds to an expected increase of 0.076 units in the CGMS score on the 5-point Likert scale (*B* = 0.076, *β* = 0.239, *p* < .001), making it one of the strongest associations in the model. The relationship of “social media effect” (X_2_) on cinema-going decision shows that a one-unit increase in this variable is associated with a 0.064-point increase in CGMS, but this association is weaker than that of “film posters”. Variables such as watching films as an artistic activity, watching films for relaxation, watching films to discuss with one’s social circle, and preferring cinema as a film-watching venue have relatively low to moderate but statistically significant associations.

**Table 7:** Predictors of CGMS.

Model Summary	R	R^2^		Adj. R^2^		SE of the Est.		Durbin-Watson	
	0.595	0.354		0.350		0.59517		1.968	
						95% CIs			
Coefficients	B	SE	β	t	p	Lower Bound	Upper Bound	Tolerance	VIF
(Constant)	1.144	0.109		10.538	.000	[.931]	[1.357]		
(X_1_) Level of interest in cinema	0.076	0.009	0.239	8.023	.000	[.057]	[.095]	0.731	1.369
(X_2_) Influence of social media on cinema-going decision	0.064	0.018	0.094	3.483	.001	[.028]	[.100]	0.887	1.127
(X_3_) Influence of film posters on cinema-going decision	0.076	0.018	0.115	4.165	.000	[.040]	[.112]	0.844	1.185
(X_4_) Watching films as an artistic activity	0.057	0.018	0.094	3.203	.001	[.022]	[.092]	0.748	1.337
(X_5_) Watching films for relaxation	0.096	0.020	0.131	4.709	.000	[.056]	[.137]	0.840	1.190
(X_6_) Watching films to discuss with social circle	0.077	0.017	0.127	4.460	.000	[.043]	[.110]	0.796	1.257
(X_7_) Preference for cinema as a film-watching venue	0.173	0.023	0.212	7.448	.000	[.127]	[.219]	0.794	1.260

Y (CGMS)=1,144+0,076*X_1_ +0,064*X_2_ +0,076*X_3_ +0,057*X_4_ +0,096*X_5_ +0,077*X_6_ +0,173*X_7_ + ε

The model in Table 7 was constructed to identify predictors of cinema-going motivation. However, as it was previously noted, motivation does not always have a decisive role on actual cinema attendance. Therefore, an ordinal regression model was conducted to explore which variables are associated with the frequency of cinema attendance. Table 8 summarizes the results of this model, which predicts the number of cinema attendance in the last 2–3 years. This model, like the multiple linear regression model, was constructed using a stepwise variable selection procedure. Based on the significance levels of the independent variables (p < .05), only those demonstrating statistically significant relationships were included in the final model. In this model, the dependent variable is the number of cinema visits over the past 2–3 years, and the independent variables are (i) level of interest in cinema, (ii) preference for cinema as a film-watching venue, (iii) film-viewing location, (iv) monthly personal spending, (v) family income, and (vi) film preference. Before addressing the results of the analysis conducted between the relevant variables, the proportional odds (parallel lines) assumption—the key assumption of the ordinal regression model—was tested. The test result was found to be statistically non-significant (χ²(66) = 75.415, p = .200). This indicates that the assumption was not violated and that the ordinal regression model established is valid.

**Table 8:** Summary of the regression model with variables predicting the frequency of cinema attendance.

	Model Fitting Information				Goodness-of-Fit				Pseudo R-Square	
Model	−2 Log Likeli.	χ^2^	df	p		χ^2^	df	p	Cox & Snell	.394
Intercept Only	2978.245				Pearson	3997.514	3754	.003	Nagelkerke	.420
Final	2409.411	568.834	22	.000	Deviance	2265.266	3754	1.000	McFadden	.180

When examining the model fit information, the initial −2 Log Likelihood value was 2978.245. After including the independent variables in the model, this value significantly improved to 2409.411 (χ² = 568.834, p < .05), indicating that the model is statistically significant. Regarding goodness-of-fit, the Pearson χ² value was 3997.514 and statistically significant (p = .003). Normally, a nonsignificant Pearson χ² is expected because this statistic assesses the model’s fit to the data, and a significant value suggests poor fit. However, this outcome is common in ordinal regression analyses and does not entirely invalidate the model’s usability. Moreover, Pearson χ² is known to be sensitive to sample size; in large samples, even minor discrepancies may become statistically significant [57]. Given that this study’s dataset includes 1,181 participants, it is reasonable to assume the large dataset influenced the significance of the Pearson χ². Another indicator of model fit, the Deviance χ², was 2265.266 (p = 1.000), showing a nonsignificant result and thus supporting good model fit. Finally, all independent variables together explained 42% of the variance in the dependent variable (Nagelkerke R² = .420). This means that the number of cinema visits in the last 2–3 years is predicted at a 42% rate by six variables: (i) level of interest in cinema, (ii) preference for cinema as a film-watching venue, (iii) film-viewing location, (iv) monthly personal expenditure, (v) family income, and (vi) film preference.

Table 9 presents the parameter estimates for the ordinal regression model. Threshold parameters represent the cut-points separating categories of the dependent variable and do not indicate the magnitude of predictor effects. The effects of the independent variables are reflected in the location coefficients (β values), which are assumed to be constant across all threshold levels under the proportional odds assumption. Furthermore, a one-unit increase in the variable “Level of Interest in Cinema” corresponds to a 0.212-unit increase in the log-odds of attending the cinema more frequently (*W* = 52.905, *p* < .001), corresponding to a 23.6% increase in the odds. This finding indicates that a higher interest in cinema is positively associated with greater odds of more frequent cinema attendance. Additionally, the frequency of cinema attendance also tends to increase as the importance placed on “preferring the cinema as a film-watching venue” increases. This variable appears to have a stronger association, as reflected by its larger coefficient.

**Table 9:** Parameter estimates of the ordinal regression model.

		Estimate	SE	Wald	df	p	95% CIs	
							Lower Bound	Upper Bound
Threshold	Number of cinema visits in last 2–3 years = Never attended	−0.502	.389	1.662	1	.197	−1.265	0.261
	Number of cinema visits in last 2–3 years = 1–3]	2.611	.397	43.304	1	.000	1.833	3.389
	Number of cinema visits in last 2–3 years = 4–6]	4.118	.407	102.333	1	.000	3.320	4.916
	Number of cinema visits in last 2–3 years = 7–10]	5.474	.423	167.642	1	.000	4.646	6.303
Location	Level of interest in cinema	0.212	.029	52.905	1	.000	0.155	0.269
	Preference for cinema as afilm-watching venue	0.750	.077	95.178	1	.000	0.599	0.900
	[Film-viewing location= I do not attend cinema, I mostly watch films on TV]	−2.081	.329	40.110	1	.000	−2.726	−1.437
	[Film-viewing location= I do not attend cinema, I mostly watch films on the internet]	−1.188	.167	50.446	1	.000	−1.516	−0.860
	[Film-viewing location= I do not attend cinema, I mostly watch films on DVD]	−0.142	.667	0.46	1	.831	−1.450	1.165
	[Film-viewing location= I do not attend cinema, I mostly watch films on digital platforms]	−0.863	.168	26.325	1	.000	−1.192	−0.533
	[Film-viewing location= I attend cinema, I mostly watch films in cinema]	0.049	.209	0.055	1	.815	−0.360	0.458
	[Film-viewing location= I attend cinema, I mostly watch films both in cinema and on digital platforms, internet, DVD]	0^a^	.	.	0	.	.	.
	[Monthly spending=5.000 TL or less]	−0.442	.263	2.821	1	.093	−0.957	0.074
	[Monthly spending =5.000-10.000 TL]	−0.259	.244	1.121	1	.290	−0.737	0.220
	[Monthly spending =10.000- 15.000 TL]	−0.253	.265	0.912	1	.340	−0.773	0.267
	[Monthly spending =15.000-20.000 TL]	0.029	.302	0.009	1	.924	−0.562	0.620
	[Monthly spending =20.000-25.000 TL]	0.178	.369	0.234	1	.629	−0.545	0.902
	[Monthly spending =25.000 TL or more]	0^a^	.	.	0	.	.	.
	[Family income=15.000 TL or less]	−1.149	.339	11.477	1	.001	−1.813	−0.484
	[Family income =15.000-30.000 TL]	−0.689	.300	5.287	1	.021	−1.277	−0.102
	[Family income =30.000-45.000 TL]	−0.449	.299	2.255	1	.133	−1.036	0.137
	[Family income =45.000- 60.000 TL]	−0.433	.302	2.050	1	.152	−1.026	0.160
	[Family income =60.000- 75.000 TL]	−0.314	.313	1.005	1	.316	−0.928	0.300
	[Family income =75.000-90.000 TL]	−0.438	.333	1.727	1	.189	−1.091	0.215
	[Family income =90.000- 105.000TL]	−0.179	.337	0.282	1	.595	−0.839	0.481
	[Family income =105.000- 120.000TL]	0.150	.464	0.105	1	.746	−0.760	1.061
	[Family income =120.000- 135.000TL]	0.364	.466	0.610	1	.435	−0.550	1.278
	[Family income =135.000 TL or more]	0^a^	.	.	0	.	.	.
	[Film preference=Domestic]	−0.461	.129	12.809	1	.000	−0.713	−0.208
	[Film preference=Foreign]	0^a^	.	.	0	.	.	.

^a^This parameter is set to zero because it is redundant.

The independent variable “film-viewing location” also yields expected results. Individuals who do not go the cinema and instead watch films on television tend to be significantly less likely to go to the cinema. Those who watch films on the internet also tend to go to the cinema less frequently, although this association is weaker compared to those who watch films on television. Similarly, individuals who use digital platforms to watch films show a decreased tendency to go to the cinema. When examining the “monthly spending” variable, no significant differences are observed compared to the reference category (25,000 TL and above). However, individuals with monthly spending of 15,000 TL or less have a lower likelihood of going to the cinema. Moreover, a positive association is observed between monthly spending and cinema attendance within this sample, although this relationship is not statistically significant. A similar pattern is observed for the “family income” variable. Those with family incomes of 15,000 TL or less and between 15,000–30,000 TL tend to attend the cinema significantly less. A positive association is observed between family income and cinema attendance within this sample, although this relationship is not statistically significant. Finally, when examining the “film preference” variable, it is found that individuals who prefer domestic films attend the cinema significantly less than those who prefer foreign films.

When interpreting these findings, the exploratory nature of the analyses should be taken into consideration. Both the multiple linear regression and ordinal regression models were constructed using a data-driven approach and are not based on strong, pre-established theoretical models. Therefore, the results should be interpreted as exploratory findings intended to generate hypotheses for future research, rather than as definitive causal inferences. In particular, terms such as ‘determinants’ or ‘predictors’ are used here in a statistical sense and do not imply causation. The findings should be interpreted within the context of the present sample, without claiming generalizability to the broader population.

## Discussion and conclusion

A significant portion of research conducted in the field of cinema in Türkiye has examined audience cinema-going habits, intergenerational transformations and the structural changes within cinema venues [8,9,20–23,25]. This study contributes to the literature not only by analyzing habits but also by evaluating young people’s motivations for going to the cinema. Moreover, by approaching cinema-going behavior from a class-based perspective, it highlights a dimension that has received limited attention in the existing literature. The findings corroborate earlier studies showing that cinema-going in Türkiye is imbued with different meanings by individuals and is often perceived as a social activity undertaken with friends or romantic partners and within positive emotional contexts [3,23,24]. Moreover, this study extends the literature in ways that both align with and diverge from previous findings. For example, international research has frequently associated cinema-going motivations with factors such as escapism, alleviating loneliness, and emotional regulation [32,33,38–42]. Yet the results of the Cinema-Going Motivation Scale (CGMS) developed in this study reveal that loneliness-related motivations are notably weak, whereas motivations related to social interaction and technical–aesthetic gratification are dominant. This divergence suggests that young people may now be fulfilling their needs for escapism or relief from loneliness primarily through digital platforms, while cinema increasingly functions as a selective, socially-oriented activity. Türkiye’s post-digital media environment differs markedly from earlier periods in this respect.

An examination of the subdimensions of the scale shows that the strongest motivations derive from “the technical features of cinema” and the related dimension of “aesthetic/emotional appeal”. The single most dominant item is “I enjoy spending time with my friends”. This clearly indicates that social interaction is the primary motivation for going to the cinema. In contrast, the low scores for loneliness-related items show that cinema-going is not perceived as an individual form of escape, but rather as a shared, collective experience.

The study also identifies a small group of participants who appear to treat cinema as a central leisure practice. These individuals report attending the cinema regardless of their mood or companions and describe cinema as an important part of their everyday routines. This finding aligns with Bourdieu’s conceptualization of cultural capital: preferences and practices related to cinema become embedded within one’s habitus as enduring cultural dispositions.

The cluster analysis conducted in the study offers important insights into how digitalization and class shape cinema-going behavior. Approximately one-third of participants are classified as “cinema-goers despite digitalization”. This group attends the cinema most frequently, places high value on its technical qualities and demonstrates tendencies associated with middle–upper or upper classes. Conversely, approximately one-fifth of the participants (22.2%) are classified as “radical digitalizers” (labels used here as heuristic descriptors based on the dominant tendencies within each group), who have not attended a cinema at all in the past 2–3 years and have shifted their viewing habits entirely to Over-The-Top (OTT) platforms. The largest group, comprising nearly half of all participants (44.8%), consists of the “moderates”, who, attend the cinema occasionally but consume most films at home. The rise in ticket prices, inflationary pressures and the widespread use of digital platforms in Türkiye’s contemporary media market appear to be associated with the formation of these clusters.

Historically, cinema has been known as a low-cost mass entertainment form [4,12,18,43,44]. However, this study suggests, at least within the sampled population of university students in contemporary Türkiye, cinema is becoming an increasingly stratified cultural participation practice, with socioeconomic indicators appearing to shape access and frequency within this sample. Within this sample, factors such as income, parental education, and personal spending capacity show meaningful associations with cinema-going behavior, though causal interpretations are not warranted given the cross-sectional design. This finding is consistent with Bourdieu’s argument that economic and cultural capital exert a determining influence on cultural participation [47].

Among the participants in this study, the capacity to translate motivation into action appears to be associated with socioeconomic position. To understand cinema-going practices in the digital age, psychological motivations must be considered alongside social position, cultural capital, and economic means. By integrating Bourdieu’s cultural capital approach with the Uses and Gratifications framework for the first time, the study offers an original theoretical contribution to the distinction between motivation and behavior.

The findings lead to several practical recommendations for cinema operators, distributors, and cultural policymakers:

1. Price differentiation (e.g., student days, income-sensitive pricing) may reduce class-based barriers.
2. Since the strongest motivation is socializing with friends, group tickets or “friend packages” may be effective.
3. As technical quality is a key motivator, investment in technical equipment should be emphasized.
4. University–campus partnerships could help attract “moderate” young digital audiences back to cinemas.
5. As trailers and social media correlate directly with motivation, digital marketing strategies should be strengthened.
6. At a time when cultural participation is becoming more unequal, support from local authorities, access programs for students, or municipal cinemas can enhance social inclusion.

The study has several limitations. First, the sample consists solely of university students recruited through convenience sampling across 12 institutions. Therefore, the findings cannot be generalized beyond this student population; non-student adults, older age groups, individuals with lower levels of formal education, and rural populations are not represented in the data. Second, the data were collected through self-reporting and may contain recall or social desirability bias. Third, the study is cross-sectional, preventing causal inferences between variables. This limitation applies not only to the regression findings but also to the cluster-based and class-related interpretations, which should be read as descriptive associations rather than causal or explanatory relationships. Fourth, although the CGMS has undergone extensive validity testing, it should be revalidated with different age groups and more diverse samples. Fifth, the cluster analysis is exploratory in nature and carries inherent limitations both in terms of the variables included and the resulting cluster structures. The clusters identified in this study do not represent sharply bounded categories; within-group diversity exists and the boundaries between groups are permeable. Furthermore, the silhouette coefficient of 0.20 indicates only a fair level of cluster separation. Future research is therefore encouraged to replicate these cluster structures using a broader and more analytically diverse set of variables, as well as larger and more representative samples. Sixth, class was operationalized through indirect proxy measures — namely income, personal expenditure, parental education level, and university type. Although these indicators provide a useful approximation, they do not fully capture the multidimensional nature of class as conceptualized by Bourdieu, which encompasses embodied dispositions, cultural tastes, and field-specific forms of capital. Accordingly, the class-related patterns reported here should be treated as socioeconomic tendencies observed within this sample rather than as evidence of definitive class positions.

Finally, the regression models were developed using an exploratory, data-driven approach in the absence of a strongly validated theoretical model in this domain. The findings should therefore be interpreted as hypothesis-generating rather than confirmatory and replicated in future studies with theoretically grounded model specifications.

Future studies should aim to validate the CGMS using larger and more diverse samples, test potential mediation/moderation models (e.g., class → motivation → participation), conduct mixed-methods investigations for deeper insight into audience experience, and integrate market data (ticket prices, spatial accessibility, OTT usage statistics) into analysis. Longitudinal research examining the long-term effects of inflation, competition among digital platforms, and changes in cultural policy would also be valuable.

This study provides clear answers to four research questions. (RQ1) Motivations cluster around social interaction and technical–aesthetic gratification, while themes of loneliness and escapism remain weak. (RQ2) Although habits have shifted strongly toward digital platforms, a distinct group continues to sustain cinema culture. (RQ3) Cinema-going behavior displays socioeconomic patterns within this sample, with participants reporting higher income and parental education levels tending to attend more frequently. (RQ4) Motivations align with aesthetic, social, and relaxation-based preferences, whereas actual participation is associated with economic indicators, film-viewing location, and general interest in cinema. Taken together, the findings demonstrate that while motivations remain relatively stable, the capacity to act on those motivations is shaped by socioeconomic position, economic conditions, and Türkiye’s increasingly digital media environment.
